# Supplementary material for: Environmental health literacy competencies and teaching methods for students and health professionals in Primary Health Care: A scoping review
Source: PLoS One. 2026 Apr 10;21(4):e0343112. doi: 10.1371/journal.pone.0343112 (PMC13068257; doi:10.1371/journal.pone.0343112)
Supplement: S1 File — (DOCX) [file pone.0343112.s001.docx]

**1. Medline:**

**Search strategy:**

| **Search conducted on October 28, 2025** | **Results** |
| --- | --- |
| ((((("professional competence"[MeSH Terms] OR Professional Competence[Text Word]) OR ("knowledge"[MeSH Terms] OR Knowledge[Text Word])) OR ("attitude"[MeSH Terms] OR Attitude[Text Word])) OR ("attitude of health personnel"[MeSH Terms] OR Attitude of Health Personnel[Text Word])) AND (("environmental health"[MeSH Terms] OR Environmental health[Text Word]) OR ("Planetary Health"[Title/Abstract] OR ("Environmental Health Literacy"[Title/Abstract] OR "Climate Health Literacy"[Title/Abstract] OR "Climate-specific Health Literacy"[Title/Abstract])))) AND ("Primary Health Care"[MeSH Terms] AND "primary care"[Title/Abstract) | 18 |
| ("professional competence"[MeSH Terms] OR "professional competence"[Text Word] OR ("Knowledge"[MeSH Terms] OR "Knowledge"[Text Word]) OR ("Attitude"[MeSH Terms] OR "Attitude"[Text Word]) OR ("attitude of health personnel"[MeSH Terms] OR "attitude of health personnel"[Text Word])) AND ("environmental health"[MeSH Terms] OR "environmental health"[Text Word] OR ("Planetary Health"[Title/Abstract] OR ("Environmental Health Literacy"[Title/Abstract] OR "Climate Health Literacy"[Title/Abstract] OR "Climate-specific Health Literacy"[Title/Abstract]))) AND ("Heath Personnel"[Title/Abstract] OR "health personnel"[MeSH Terms] OR "health personnel"[Text Word] OR "health occupations"[MeSH Terms] OR "health occupations"[Text Word] OR "allied health personnel"[MeSH Terms] OR "allied health personnel"[Text Word] OR "Healthcare Professionals"[Title/Abstract] OR "Healthcare Providers"[Title/Abstract] OR "Health Workers"[Title/Abstract] OR "nurses"[Title/Abstract] OR "physicians"[Title/Abstract] OR "dentists"[Title/Abstract] OR "pharmacists"[Title/Abstract] OR "Public Health Professionals"[Title/Abstract]) | 3.228 |
| **Total** | **3.246** |

**2. Web of Science:**

**Search strategy:**

| **Search conducted on October 29, 2025** | **Results** |
| --- | --- |
| (((TI=("Primary Health Care")) OR AB=("Primary Health Care")) OR TI=("primary care")) OR AB=("primary care")  AND  (((((((TI=("environmental health")) OR AB=("environmental health")) OR TI=("Environmental Health Literacy")) OR AB=("Environmental Health Literacy")) OR TI=("Climate Health Literacy")) OR AB=("Climate Health Literacy")) OR TI=("Planetary Health")) OR AB=("Planetary Health") AND ((((((((TI=("professional competence")) OR AB=("professional competence")) OR TI=(knowledge)) OR AB=(knowledge)) OR TI=(attitude)) OR AB=(attitude)) OR TI=("Attitude of Health Personnel")) OR AB=("Attitude of Health Personnel")) | 119 |
| **Total** | 119 |

**3. Embase:**

**Search strategy:**

| **Search conducted on October 29, 2025** | **Results** |
| --- | --- |
| ('medical profession'/exp OR 'health occupations' OR 'medical profession' OR 'health care personnel'/exp OR 'health care personnel' OR 'health care practitioner' OR 'health care professional' OR 'health care provider' OR 'health care worker' OR 'health personnel' OR 'health profession personnel' OR 'health worker' OR 'healthcare personnel' OR 'healthcare practitioner' OR 'healthcare professional' OR 'healthcare provider' OR 'healthcare worker' OR 'home health aides' OR 'personnel, health' OR 'public health officer' OR 'paramedical personnel'/exp OR 'allied health personnel' OR 'health care assistant' OR 'health care support worker' OR 'health support worker' OR 'healthcare assistant' OR 'healthcare support worker' OR 'ophthalmic assistants' OR 'para medical personnel' OR 'paramedical assistant' OR 'paramedical manpower' OR 'paramedical personnel' OR 'paramedical professional' OR 'paramedical staff' OR 'paramedics' OR 'psychiatric aides') AND ('environmental health'/exp OR 'environmental health' OR 'health, environmental' OR 'environmental health literacy':ti,ab OR 'climate health literacy':ti,ab OR 'planetary health'/exp OR 'health of our planet' OR 'health of the planet' OR 'planet health' OR 'planet`s health' OR 'planetary health' OR 'planetary well-being' OR 'planetary wellbeing') AND ('professional competence'/exp OR 'professional competence' OR 'knowledge'/exp OR 'knowledge' OR 'health personnel attitude'/exp OR 'attitude of health care professionals' OR 'attitude of health care workers' OR 'attitude of health personnel' OR 'attitude of health professionals' OR 'attitude of healthcare professionals' OR 'attitude of healthcare workers' OR 'health care personnel attitude' OR 'health care professionals attitude' OR 'health care staff attitude' OR 'health personnel attitude' OR 'health professionals attitude' OR 'health staff attitude' OR 'healthcare personnel attitude' OR 'healthcare professionals attitude' OR 'healthcare staff attitude') AND ('primary health care'/exp OR 'first line care' OR 'health care, primary' OR 'primary care nursing' OR 'primary health care' OR 'primary healthcare' OR 'primary nursing care' OR 'primary care':ti,ab) | 108 |
| **Total** | 108 |

**4. Scopus:**

**Search strategy:**

| **Search conducted on October 29, 2025** | **Results** |
| --- | --- |
| ( ( ( TITLE-ABS-KEY ( "environmental health" ) OR TITLE-ABS-KEY ( "Planetary Health" ) OR TITLE-ABS-KEY ( "Climate Health Literacy" ) OR TITLE-ABS-KEY ( "Climate-specific Health Literacy" ) ) ) AND ( ( TITLE-ABS-KEY ( "professional competence" ) OR TITLE-ABS-KEY ( knowledge ) OR TITLE-ABS-KEY ( attitude ) OR TITLE-ABS-KEY ( "attitude of health personne" ) ) ) ) AND ( ( TITLE-ABS-KEY ( "health occupations" ) OR TITLE-ABS-KEY ( "health personnel" ) OR TITLE-ABS-KEY ( "allied health personnel" ) ) ) AND ( ( TITLE-ABS-KEY ( "Primary Health Care" ) OR TITLE-ABS-KEY ( "primary care" ) ) ) | 13 |
| ( ( ( TITLE-ABS-KEY ( "environmental health" ) OR TITLE-ABS-KEY ( "Planetary Health" ) OR TITLE-ABS-KEY ( "Climate Health Literacy" ) OR TITLE-ABS-KEY ( "Climate-specific Health Literacy" ) ) ) AND ( ( TITLE-ABS-KEY ( "professional competence" ) OR TITLE-ABS-KEY ( knowledge ) OR TITLE-ABS-KEY ( attitude ) OR TITLE-ABS-KEY ( "attitude of health personne" ) ) ) ) AND ( ( TITLE-ABS-KEY ( "Primary Health Care" ) OR TITLE-ABS-KEY ( "primary care" ) ) ) | 86 |
| **Total** | 99 |

**5. CINAHL:**

**Search strategy:**

| **Search conducted on October 29, 2025** | **Results** |
| --- | --- |
| (TI ("primary health care") OR AB ("primary health care") OR SU ("primary health care") OR (primary health care or primary care or public health care or community care)) AND ((MH "Environmental Health" OR MH "Environmental Health" OR MH "Environmental Health" OR (MH "Environmental Health" AND MH "Literacy") OR (MH "Environmental Health" AND MH "Literacy") OR (MH "Environmental Health" AND MH "Literacy") OR TI ("planetary health") OR AB ("planetary health") OR SU ("planetary health")) AND (TI ("professional competence") OR AB ("professional competence") OR SU ("professional competence") OR TI (knowledge) OR AB (knowledge) OR SU (knowledge) OR TI ("attitude of health personnel") OR AB ("attitude of health personnel") OR SU ("attitude of health personnel"))) | 152 |
| **Total** | 152 |

**5. PsycInfo:**

**Search strategy:**

| **Search conducted on October 29, 2025** | **Results** |
| --- | --- |
| ((((**title**: (professional competence))) *OR* ((**abstract**: (professional competence))) *OR* ((**title**: (knowledge))) *OR* ((**abstract**: (knowledge))) *OR* ((**title**: (attitude))) *OR* ((**abstract**: (attitude)))) *AND* (((**title**: (environmental health))) *OR* ((**abstract**: (environmental health))) *OR* ((**title**: (Environmental Health Literacy))) *OR* ((**abstract**: (Environmental Health Literacy))) *OR* ((**title**: (Planetary Health))) *OR* ((**abstract**: (Planetary Health))) *OR* ((**title**: (Climate Health Literacy))) *OR* ((**abstract**: (Climate Health Literacy))))) *AND* ((**title**: (Primary Health Care)) *OR* (**abstract**: (primary health care)) *OR* (**title**: (primary care)) *OR* (**abstract**: (primary care))) | 81 |
| **Total** | 81 |

**6. LILACS:**

**Search strategy:**

| **Search conducted on October 29, 2025** | **Results** |
| --- | --- |
| (((("Saúde Ambiental") OR ("Educação em Saúde Ambiental")) OR (("Saúde Ambiental") AND ("Letramento em Saúde"))) AND (("Competência Profissional") OR (conhecimento) OR ("Atitude do Pessoal de Saúde") OR (atitude))) AND (("Atenção Primária à Saúde") OR ("Atenção Primária Ambiental")) AND instance:"lilacsplus" | 7 |
| **Total** | 7 |

**Total records identified across databases: 3,842
Duplicate records removed: 308
Records screened: 3,534**

**Grey literature**

**OpenGrey Results**

| **Search conducted on October 29, 2025** | **Results** |
| --- | --- |
| <https://lifesciences.datastations.nl/dataverse/root/?q=%22Environmental+Health+Literacy%22+professional+competence> | 130 |
| **Total** | 130 |

**Title-based selection:** 0 records selected

**Google Scholar Results**

| **Search conducted on October 29, 2025** | **Results** |
| --- | --- |
| <https://scholar.google.com.br/scholar?as_q=professional+competence&as_epq=Environmental+health+literacy&as_oq=%22health+personnel%22+%22health+occupations%22+%22Health+Workers%22+&as_eq=&as_occt=any&as_sauthors=&as_publication=&as_ylo=&as_yhi=&hl=pt-BR&as_sdt=0%2C5> | 190 |
| <https://scholar.google.com.br/scholar?start=60&q=professional+competence+%22Environmental+health+literacy%22&hl=pt-BR&as_sdt=0,5> | 162 |
| **Total** |  |

**Title-based selection:** 4 records

**ProQuest Dissertations & Theses e repositórios institucionais**

| **Search conducted on October 29, 2025** | **Results** |
| --- | --- |
| https://www.proquest.com/resultsol/2B5F2049F1F04E09PQ/1 | 94 |
| **Total** | 94 |

**Title-based selection:** 0 records selected
